# State Medical Society

**Published:** 1858-06

**Authors:** 


### STATE MEDICAL SOCIETY.

A meeting of this society met on the 9th of June at Mount Pleasant, Henry county. Owing to the impassable condition of the roads, and the overflowing streams, the attendance was but meagre, being composed of those residing in the thriving town in which it convened, those living in towns upon the Mississippi river, and others, upon railroads reaching from the river into the interior. Dubuque, Davenport, Burlington and Keokuk, with Iowa City, Salem and Fairfield, were those only from which there were representatives. From this place there was but one delegate, (Prof. M'Gugin), owing to the fact that the criminal court was then in session, to which several members of the profession were summoned as witnesses to testify in several capital cases.

Our representative reports that, although the attendance was slim, it was nevertheless interesting. It is true, as well as unfortunate for the objects and designs of the organization, that there was so little reported, or said, upon medical topics. We trust this will not obtain in the future, but that each committee will come up prepared to report upon the subject-matters assigned to its members, and that others not on committees will contribute to the fund of useful information. The delegate from this place speaks in the highest terms of commendation of the attention and courtesies extended to the members by the medical gentlemen and the citizens and ladies of the thriving and beautiful town of Mt. Pleasant. An elegant supper was served up at the "Brazleton House," to which the members, with a number of ladies, sat down. After they had partaken, Dr. Hambline was chosen to preside, a number of sentiments were read, and quite a number of speeches made. The first toast was in honor of our State, to which Gov. Lowe, who was an invited and an honored guest, made some very happy and pertinent remarks. Mr. Elliott, the able editor of a weekly paper, also delighted the company with many witty and pointed hits.

Prof. Rauch, of Burlington, was made President of the society, and Dr. Baker, of Davenport, Recording Secretary. The next meeting will be held in Davenport.

